# Intersectoral Collaboration: What Works and What Doesn’t

**DOI:** 10.34172/ijhpm.2023.8090

**Published:** 2023-05-03

**Authors:** Elizabeth H. Bradley

**Affiliations:** Vassar College, Poughkeepsie, NY, USA.

## Dear Editor,

 The paper by Turner and colleagues is an inspiring take on how cross-sectoral collaboration can be accomplished, in the context of the jointly shared emergency of the COVID-19 pandemic. The authors review the experience of three cities in Columbia and their efforts to share knowledge and resources as well as coordinate responses to COVID-19 across governmental healthcare organizations, private sector institutions, and universities. Because these entities are typically thought to differ mission, incentives, and objectives, this careful examination of factors that enabled or impeded coordination generated novel insights that may be helpful in less urgent but still pressing healthcare needs in the future.

## Factors Enabling Cross-sectoral Coordination

 The authors highlighted factors that enabled cross-sectoral coordination across government, private, and university sectors including (1) formal spaces of collaboration, such as joint committees set up by secretariats of health, (2) long-standing and pre-existing relationships between private sector actors and economic leaders that were mobilized, and (3) complementarity in capacities such as the universities had scientific knowledge and laboratories that could be leveraged for COVID-19 testing while private sector actors had access to alliances and economic resources, and government agencies could act as conveners and representatives of the public.

## Barriers to Cross-sectoral Coordination

 Nevertheless, barriers to collaboration were documented as well. First, timelines for research differed with healthcare policy-makers needing evidence on a faster timeline than academics were accustomed to producing. Second, lack of clarity in communication between government agencies and university laboratories was challenging, particularly in the dynamic circumstances of quickly changing understandings of the virus. Third, lack of trust across sectors had to be overcome, and some participants reported challenges in lack of leadership from local government, especially at the start of the pandemic. Last, limited resources was a persistent barrier, and collaborations with the private sector entities helped in this area.

## Study Limitations

 Turner and colleagues are to be congratulated for a timely and innovative paper; however, readers would benefit from a more diverse sample of cities — perhaps including some cities that were more successful and others that were less successful in their COVID-19 response. The current paper reported on three cities as case studies but was unable to leverage variation in cities’ success to derive hypotheses about how differences in cross-sector coordination may be linked to differences in pandemic responses. Furthermore, the ability to determine from this paper “what works” in fostering effective partnerships for healthcare responses was limited, as the size and diversity of the sample were modest.

## Health Benefits of Cross-sectoral Coordination

 Despite these limitations, the findings are compelling and remind readers that coordination across sectors can confer health benefits. Similar conclusions have been found in studies documenting how some US communities achieve lower healthcare utilization and costs^1,2^ as well as how some US counties have been able to limit obesity rates despite high state-wide obesity rates.^3^ This work has found that higher performing communities (in terms of lower hospitalization rates and costs and lower obesity rates) also have had superior coordination between healthcare and social service sectors. Consistent with Turner and colleagues,^4^ this work^1-3^ has also showed the importance of pre-existing relationships, strong norms of association, and neutral or “backbone” organizations that have been trusted and able to effectively convene a range of institutions across sectors.

 The implications of this body of research are crucial for finding creative solutions to not only unexpected health crises such as COVID-19 but also for the everyday health and healthcare problems we face as a globe — including overmedicalization of health challenges, the balkanization of medicine and public health,^5^ and the persistent health disparities brought on by social, economic, and environmental determinants of health. Clearly economic resources are important, and greater health investments are paramount. Nevertheless, the study by Turner and colleagues^4^ suggests other interventions may be crucial as well—that is, the fostering of partnerships and meaningful collaboration across sectors that influence or are influenced by the population’s health. Moreover, based on the findings by Turner and colleagues, such partnerships are facilitated by pre-existing relationships, interactions, and trust. Thus, pursuing such collaborations — particularly among government, private sector, and universities — *before* the next pandemic may generate benefits not only for future pandemic response but also health policy and management decision making more broadly.

## Ethical issues

 Not applicable.

## Competing interests

 Author declares that she has no competing interests.
